# Recommendations made by patients, caregivers, providers, and decision-makers to improve transitions in care for older adults with hip fracture: a qualitative study in Ontario, Canada

**DOI:** 10.1186/s12877-022-02943-6

**Published:** 2022-04-07

**Authors:** Lauren Cadel, Kerry Kuluski, Amanda C. Everall, Sara J. T. Guilcher

**Affiliations:** 1 Leslie Dan Faculty of Pharmacy, University of Toronto, Toronto, ON Canada; 2 Institute for Better Health, Trillium Health Partners, Mississauga, ON Canada; 3 Institute of Health Policy, Management, and Evaluation, University of Toronto, Toronto, ON Canada

**Keywords:** Qualitative, Canada, Patient transfer, Delivery of health care, Quality of health care, Transitions in care

## Abstract

**Background:**

Older adults frequently experience fall-related injuries, including hip fractures. Following a hip fracture, patients receive care across a number of settings and from multiple different providers. Transitions between providers and across settings have been noted as a vulnerable time, with potentially negative impacts. Currently, there is limited research on how to improve experiences with transitions in care following a hip fracture for older adults from the perspectives of those with lived experienced. The purpose of this study was to explore service recommendations made by patients, caregivers, healthcare providers, and decision-makers for improving transitions in care for older adults with hip fracture.

**Methods:**

This descriptive qualitative study was part of a larger longitudinal qualitative multiple case study. Participants included older adults with hip fracture, caregivers supporting an individual with hip fracture, healthcare providers, and decision-makers. In-depth, semi-structured interviews were conducted with all participants, with patients and caregivers having the opportunity to participate in follow-up interviews as they transitioned out of hospital. All interviews were audio-recorded, transcribed verbatim, and analyzed thematically.

**Results:**

A total of 47 participants took part in 65 interviews. We identified three main categories of recommendations: (1) hospital-based recommendations; (2) community-based recommendations; and (3) cross-sectoral based recommendations. Hospital-based recommendations focused on treating patients and families with respect, improving the consistency, frequency, and comprehensiveness of communication between hospital providers and between providers and families, and increasing staffing levels. Community-based recommendations included the early identification of at-risk individuals and providing preventative and educational programs. Cross-sectoral based recommendations were grounded in enhanced system navigation through communication and care navigators, particularly within primary and community care settings.

**Conclusions:**

Our findings highlighted the central role primary care can play in providing targeted, integrated services for older adults with hip fracture. The recommendations outlined have the potential to improve experiences with care transitions for older adults with hip fracture, and thus, addressing and acting on them should be a priority.

## Background

Fall-related injuries, including fractures, are common as the population ages [1, 2], with hospitalizations due to hip fractures being one of the most frequent diagnoses of fall-related injuries [3, 4]. Hip fractures are especially prevalent in North America, affecting up to 300,000 Americans and 30,000 Canadians every year [5–7]. In addition to older age, common risk factors for hip fractures include: being female, history of osteoporosis, physical inactivity, use of multiple medications, and chronic health conditions [8, 9]. Adults with hip fracture often experience hospital-associated deconditioning and delays in discharge [10], a decreased ability to perform activities of daily living [11], a decline in overall health-related quality of life [12], and high readmission rates [13].

Adults with hip fracture often receive care and support across a range of settings such as acute care hospitals, inpatient rehabilitation units, outpatient ambulatory care clinics, assisted living facilities, long-term care homes, and home with homecare [14–17]. As a result, individuals can experience a number of care transitions post-injury, which are defined as moving from one setting to another or between healthcare providers [18]. Transitions in care have been highlighted as an increased time of vulnerability for patients and caregivers, with the possibility for poor transitions resulting in readmission, medication errors, decreased patient and caregiver satisfaction, and poor health and well-being outcomes [18–20].

A scoping review by Asif and colleagues (2020) explored what is known in the literature on the experiences of patients with hip fracture and caregivers for adults with hip fracture, and noted an overall gap in research identifying the experiences and needs of patients and caregivers [21]. Of the limited research, the majority is qualitative and broadly explores experiences with transitions in care, often identifying challenges with the process. Asif and colleagues (2020) highlighted disorganized discharge planning, poor communication and information sharing, and role confusion amongst patients, caregivers, and providers as common challenges occurring during care transitions. In response to the limited research, we aimed to specifically explore service recommendations made by patients, caregivers, healthcare providers, and decision-makers (e.g. organizational leaders, managers) for improving transitions in care for older adults with hip fracture.

## Methods

### Study design

This qualitative study was part of a larger longitudinal qualitative multiple case study [22] of two diverse health regions in Ontario, Canada. The two regions were chosen based on variation in rurality, geographical setting, care delivery processes, and system performance on hip fracture care. The Standards for Reporting Qualitative Research were followed [23].

### Participants

Study participants included patients with hip fracture, caregivers, healthcare providers, and decision-makers (individuals who impacted care processes, program implementation, and daily operations). For inclusion, patients were required to have experienced a hip fracture, be at least 50 years of age, and able to communicate in English or French. Caregivers were required to be supporting an individual with a hip fracture in an unpaid capacity (e.g. assisting with activities of daily living, providing emotional support, managing medications, handling finances), be at least 18 years of age, and able to communicate in English or French. Healthcare providers and decision-makers were required to be at least 18 years of age and provide care, or impact care processes, for persons with hip fracture within Ontario, Canada.

### Setting and recruitment

This study was conducted in Ontario, within the context of the Canadian healthcare system. In Ontario, a standard for improving the quality of hip fracture care for adults aged 50 and older was developed by a Quality Standard Advisory Committee [24]. These standards provide guidance on care delivery from the emergency department until three months post-surgery. It is expected that patients are diagnosed within one hour of arriving at the hospital, receive surgery within 48 h, have their pain assessed and managed, receive the appropriate surgery, weight-bear within 24 h of surgery (as tolerated), mobilize daily post-surgery, receive screening and management for delirium and osteoporosis, receive post-operative care from an interdisciplinary team, receive tailored information, participate in interdisciplinary rehabilitation (inpatient, community, or both), and receive follow-up care from their primary care provider and orthopedic services [24].

Participants were recruited from two hospital networks, one in each health region. Purposive and theoretical sampling strategies were used [25]. Participant criteria were identified prior to starting recruitment, but data collection and analysis continued simultaneously until reaching data saturation. Healthcare providers in the two hospital networks identified patients and caregivers who met the inclusion criteria, explained the study to them, and obtained their permission for the researchers to connect with them. All patients were initially approached in-person while in hospital, and caregivers were approached in-person or by telephone. When providing consent for the first interview, patients and caregivers had the opportunity to express interest in completing follow-up interviews post-discharge (consent to participate in future interviews did not impact participation in the first interview). Interested individuals were then contacted at a future date and consented to the follow-up interviews. Healthcare providers and decision-makers were recruited through the hospital units and the research teams’ contacts. Snowball sampling was also used to increase recruitment efforts among healthcare providers and decision-makers [25]. The number of individuals who declined contact with the researchers was not tracked; no participants dropped out of the study.

### Data collection

Data were collected through in-depth, semi-structured interviews. Interviews were conducted by trained members of the research team (ACE, MSc; JL, BSc; LC, MSc) from August 2018 to July 2019 and lasted between 15 and 90 minutes. The researchers conducting the interviews received ongoing mentorship from the senior team members (SG, PhD; KK, PhD), as well as through formal and informal training. Participants had the option of completing the interview in-person or by telephone. Initial interviews with patients and caregivers were conducted while the patient was in hospital (following transition from the emergency department to the inpatient unit). Follow-up interviews were conducted with participants as patients transitioned out of hospital to their next care setting (e.g. rehabilitation, long-term care, home). Patients and caregivers could participate in up to two follow-up interviews if they had multiple transitions following their hospitalization. All interviews were audio-recorded, transcribed verbatim, and pseudonyms were assigned to all participants. Four interview guides were used to structure the conversations – one for each participant group (patients, caregivers, providers, decision-makers). The guides were designed in consultation with key stakeholders (healthcare providers and organizational leaders) and explored participants’ overall experiences with care transitions. More specifically, the guides probed on potential barriers to discharge, resources used, required, or desired (pre and post-discharge), relationships (with patient/caregiver and providers), expectations for transitions, concerns with discharge, and community and social support. The interviewer engaged in reflexive notetaking following each interview in order to make note of potential areas to probe in future interviews and to summarize key reflections.

### Data analysis

Data collection and analysis occurred simultaneously until data saturation was achieved, that is no new concepts were identified relevant to our research questions. The data analysis process was guided by the framework method [26]. As interviews were conducted and transcribed, they were reviewed and discussed in detail in weekly team meetings. An initial codebook, grounded in the data, was developed by the study team in these ongoing meetings. This codebook was applied to a subset of transcripts by three coders (ACE, JL, LC). These individuals prepared for the coding process by familiarizing themselves with the data (reading transcripts from different participant types) and the codebook (reviewing the codes, definitions, and examples). The coders’ agreement was compared (98% agreement) and minor revisions and clarifications were made to the codebook. The finalized codebook was applied to all transcripts by the coders (ACE, JL, LC). NVivo11 was used to manage and organize the coded data.

In the second phase of analysis, relevant codes (experiences with care transitions, factors that impact care transitions, local initiatives to improve care transitions, service optimization recommendations) from the codebook were exported and reviewed by the team. These codes were input into Microsoft Excel to allow for comparison. During team meetings, the codes were merged into the categories and themes presented below.

### Theoretical orientation

This research was grounded in pragmatism [27], based on our goal to inform real-world practice [28]. The pragmatism paradigm acknowledges that there can be multiple realities, or interpretations of a reality [28]. It also recognizes that the participants’ experiences are based on their personal beliefs, habits, and social interactions, and thus, each individual can have their own unique knowledge [29].

### Ethics

This study received ethics approval from the required Research Ethics Boards. All research was carried out according to the approved processes.

## Results

A total of 47 participants, including 15 patients, 15 healthcare providers, 10 caregivers, and 7 decision-makers, took part in 65 interviews. The numbers of participants and interviews were evenly distributed across the two regions. Participant characteristics are displayed in Tables 1, 2, and 3. Three main categories of recommendations were identified: (1) hospital-based recommendations; (2) community-based recommendations; and (3) cross-sectoral based recommendations (see Table 4). The recommendations made by participants were similar across both regions and thus, are presented together.

**Table 1:** Demographic characteristics of patients (*n* = 15)

Characteristic	# Patients
***Age***	
Under 65	2
65 – 85	8
Over 85	4
Unknown	1
***Sex***	
Male	6
Female	9
***Living Arrangements***	
Lives alone	10
Lives with family	5
***Rurality***	
Urban	8
Rural	7
***Discharge Location***	
Long-term care	0
Home	7
Assisted Living	0
Unknown^a^	8

^a^Discharge location is unknown for participants who did not complete a follow-up interview

**Table 2:** Demographic characteristics of caregivers (*n* = 10)

Characteristic	# Caregivers
***Caregiver Age***	
Under 65	2
65 – 85	1
Over 85	1
Unknown	6
***Caregiver Sex***	
Male	3
Female	7
***Relationship to patient***	
Spouse/ Partner	3
Child	6
Other	1
***Living arrangements in respect to patient***	
With patient	3
Same city	4
Other	3
***Age of patient***	
65 – 85	1
Over 85	6
Unknown	3
***Rurality***	
Urban	6
Rural	4
***Patient’s Discharge Location***^a^	
Long-term care	2
Home	2
Assisted Living	1
Unknown	4
Died in hospital	1

^a^Discharge location is unknown for participants who did not complete a follow-up interview

**Table 3:** Demographic characteristics of providers (*n* = 15) and decision-makers (*n* = 7)

Characteristic	# Providers	# Decision-makers
***Sex***		
Male	2	2
Female	13	5
***Years in practice***		
1 – 5 years	1	2
6 – 10 years	2	1
11 – 15 years	4	1
16 – 20 years	3	1
21 + years	4	1
Not reported	1	1
***Role***^a^		
Nurse	5	1
Allied health^b^	10	2
Physician	0	1
Senior Executive	0	1
Director	1	2
Team lead/ manager	2	4
***Rurality***		
Urban	5	3
Rural	10	4

^a^Providers and decision-makers with a clinical background and in a leadership role are counted twice in role

^b^Allied health providers consisted of physical and occupational therapists and social workers

**Table 4:** Categories and recommendations made by participants

Category	Recommendation	Recommended By
Hospital-based	Focus on people before patients – more than a number • Treat patients as individuals, show empathy and respect, have patience	Patients, caregivers, providers
	Provide consistent, frequent, and comprehensive communication • Patients and caregivers should receive the same information from all members of the patients care team • Communication should be ongoing and detailed	Patients, caregivers, providers, and decision-makers
	Increase staffing – more help is needed • More staff support from physical and occupational therapists, nurses, geriatricians, and personal support workers	Patients, caregivers, providers, and decision-makers
Community-based	Identify at risk individuals in primary health care • Increase support in the community to identify individuals who may be at risk of experiencing a hip fracture (falls, functional decline, etc.)	Providers and decision-makers
	Prevent and educate – reacting is not enough • Increase education and availability of information about supports and programs in the community (falls prevention)	Patients, caregivers, providers, and decision-makers
Cross-sectoral based	Enhance supports through care navigators • An individual to coordinate required services for the patient (physical therapy, occupational therapy, social work)	Providers and decision-makers
	Improve cross-sectoral communication • Improve communication with primary care and community-based providers	Providers and decision-makers

### Hospital-based recommendations

Hospital-based recommendations made by patients, caregivers, providers, and decision-makers were specific to any care, supports or services contained within the hospital, including acute care and inpatient rehabilitation. Three themes were identified: (1) Focus on people before patients – more than a number; (2) Provide consistent, frequent, and comprehensive communication; and (3) Increase staffing – more help is needed.

#### Focus on people before patients – more than a number

Patients and caregivers emphasized the importance of being treated as a person and “stop[ping] and look[ing] at the people you’re dealing with” [Olivia, caregiver].

*You know, we’ve been for the most part, we’ve been very lucky with people being very kind and patient. But there are a few who tend to forget that, you know, these people have value and they are people and you just need to take time and be gentle. Give them a smile and, you know. (Olivia, caregiver, follow-up)*

Caregivers described the need for providers to be “more sympathetic to the patients” [Emma﻿, caregiver], “pleasant and social” [Mason﻿, caregiver], and “have that softer side or gentler side when dealing with geriatrics” [Emma, caregiver, follow-up]. Patients echoed these recommendations, suggesting that providers show increased patience and respect, as one individual explained:

*Patience. Patience with a capital ‘P’, patience… treat that injured person or, or person like me with, with patience and respect… Show respect that you would get - that you would expect to get when you were in the bed or wherever. (Ronald, patient)*

Another patient explained the importance of being treated as an individual:

*Like you get so many people who don’t give a hoot… Just kinda maybe individualize each client, you know, each patient because even though we’ve all had whatever, hip replacements or whatever, we shouldn’t all be lumped in one big thing… Everybody’s different. (Meghan, patient, follow-up)*

#### Provide consistent, frequent, and comprehensive communication

Across all participant groups, improved communication in terms of consistency, frequency, and comprehensiveness was recommended for enhancing patient experiences in hospital. Patients and caregivers described the need for improved frequency and comprehensiveness of communication from providers, while providers mostly described the need for improved consistency of communication with patients and families and between providers. Caregivers expressed frustrations when they were not informed of major decisions regarding the patients’ care (e.g., surgery details or internal transfers).

*I’m the first contact. So, I would’ve imagined that either my father or myself would’ve got a call just to say that mother is being taken up to surgery. Nothing. (Olivia, caregiver, follow-up)*

Providers emphasized the importance of ensuring patients and caregivers were given consistent information, especially as it pertained to their care transitions. When patients received mixed messages, it often led to confusion and at times, disappointment.

*I mean just yesterday, I had to tell a patient that she would not be going to IRU (Intensive Rehabilitation Unit) because the doctor told her that she would be going to IRU which is not accurate because she’s not appropriate for IRU and (sigh), yeah. (Chloe, provider)*

This provider further explained:

*Well, consistency and yeah, consistent messaging from all of the healthcare providers, which would need education for all of the healthcare providers on what the destinations are and who can go and so, that would require buy in from the different healthcare providers on like really attending and following those like criteria and stuff. But I think consistency is like one of the biggest things and just knowing that everybody is giving the same information to the same patient. (Chloe, provider)*

#### Increase staffing – more help is needed

A key recommendation made across all participant groups was to increase staffing in hospitals, specifically physical and occupational therapists, nurses, geriatricians, and personal support workers (PSWs). Some patients experienced, firsthand, the perceived negative impact of budget cuts and staff shortages on inpatient units.

*Like I think the cutbacks, I’m sure that’s where it stems from... I know our dialysis unit lost a social worker and nurses and, you know what, it used to run like a well-oiled machine and it doesn’t anymore. You know, there’s like cogs missing. (Lauren, patient)*

Increased staffing was recommended in order to reduce provider and caregiver burnout, ensure patient calls were responded to in a timely manner, help patients get up for meals, and increase the frequency and duration of patients’ physical therapy sessions. Patients and caregivers clearly articulated this need for increased support by suggesting “… more of these people on the floors. More nurses, more PSWs [Personal Support Workers]. They need more help. That's all I can say” [Mason, caregiver, follow-up]. This statement was echoed by a decision-maker, who also described the need for more geriatricians, more providers who have had specific training in geriatric syndromes, and more general geriatric education.

*I think you need about six more geriatricians in that hospital and you need to revamp… For multi-disciplinary teams. If I’ve had to do this much work to bring geriatric best practice to one rehab unit to attack an entire hospital. Like these are not folks who were trained in geriatrics in this type of multidisciplinary care rounding approach for older adults, you know. No one’s had that training and I think there’s a huge gap in understanding of geriatric syndromes and functional decline and hospital related functional decline. (Lillian, decision-maker)*

### Community-based recommendations

Community-based recommendations made by patients, caregivers, providers, and decision-makers were specific to care, supports, or services obtained outside hospital, including long-term care and home and community care. Two themes were identified: (1) Need for identification of individuals at risk in primary health care and (2) Prevent and educate – reacting is not enough.

#### Need for identification of individuals at risk in primary health care

Providers and decision-makers described the need for increased support in the community to identify individuals who may be at risk of experiencing a hip fracture due to falls or functional decline. This support was recommended to come from family physicians through rehabilitation referrals.

*… in family physician’s office identify, wow you are failing and you’re having functional decline and there’s some reversibility here. Let's get you into rehab for two, three weeks to see if they can reverse some of this and improve your function to prevent a fall, prevent an admission [to hospital]. Getting them even before they come to hospital. (Aubrey, provider)*

The potential role of family physicians in early identification was described, explaining that not only could they help prevent initial hospital admissions, but also prevent readmissions. Providers discussed the importance of community-based providers connecting with patients following their transition to the community (home, assisted living, etc.) to ensure they were managing well and had the necessary supports following a hospitalization.

*If they’re not succeeding at home, that should be identified early… There’s gotta be somebody there [at home] fairly quickly that’s able to identify that before it becomes an issue and they’re falling or they’re not eating or, you know. And they end up at Emerg again and have to go through the whole process again. (Emily, provider)*

Patients and caregivers did not discuss any recommendations around the need for identifying at-risk individuals in the community.

#### Prevent and educate – reacting is not enough

Related to the need for increased identification of at-risk individuals, all participants described the importance of preventative education and services. Shifting the reactive model of care to a more proactive and preventative approach was a key recommendation made by participants. Some of these community-based, proactive approaches included “getting people involved in regular exercise programs and falls prevention programs” [Kerry, provider] and “more home care… More PSWs [Personal Support Workers] helping with ADLs [activities of daily living]” [Jackson, provider]. In addition to preventative programs and supports in the community, caregivers discussed the importance of proactive planning for aging family members.

*You have to, you have to be more proactive and start early on. Thinking that you have time, you don’t have time. You have to start asking questions early. Even though your parents may not be ready, you have to know what you’re up against. (Olivia, caregiver, follow-up)*

In order to be more proactive, participants described the need for increased education and availability of information about supports and programs in the community. Providers discussed community-based prevention programs that were available but acknowledged that there needed to be “more advertising of those things” [Jackson, provider]. Caregivers echoed this statement by describing the need for more education on existing supports and services available in the community and their eligibility criteria.

*… I think there’s a lack of good education for people of what’s out there… There’s a lot of things out there that a lot of people don’t know that they are entitled to unless you hear. Hearsay is how you hear about it, not by our government telling us, you know, this is available, you know. Try and find it, like the information is very poor unless somebody tells you. (Mason, caregiver)*

### Cross-sectoral based recommendations

Cross-sectoral based recommendations made by patients, caregivers, providers, and decision-makers were specific to care, supports, or services that occurred during the transitions in patients’ care journeys (hospital to long-term care, hospital to home, etc.). Two themes were identified: (1) Enhance supports through care navigators; and 2) Improve cross-sectoral communication.

#### Enhance supports through care navigators

All participant groups discussed the complex healthcare system that could be challenging to navigate, especially for older adults following a hip fracture who often had to coordinate additional supports and services to transition home. Many patients and caregivers discussed not knowing what services were available to support transitions, how to set them up, when they would start, or how long they would be available. In order to improve patients’ and caregivers’ ability to navigate the system and arrange appropriate supports, providers expressed the need for a ‘Care Navigator’, who would coordinate all required services for the patient (physical therapy, occupational therapy, social work, etc.):

*Well, yeah, if they were only dealing with one person... Navigating them through the whole thing, but because, you know, they’re dealing with a doctor, a PT [physical therapist], an OT [occupational therapist], a nurse, a social worker, myself, the rehab facility. (Aubrey, provider)*

#### Improve cross-sectoral communication

In addition to a Care Navigator role, participants expressed the need for improved communication across sectors in order to improve transitions post-hip fracture. In particular, healthcare providers discussed the lack of communication with primary care and community-based providers, which they expressed would be beneficial for collaborating and sharing contextual information about patients and their families. A hospital-based physical therapist described a lack of communication with the community sector, with most interactions limited to discharge reports, further recommending increased cross-sectoral collaboration to improve transitions from hospital to the community:

*… but no unfortunately, we don’t speak directly to the PTs [physical therapists in the community]… I mean, they would be in a great position to speak to them but we don’t, we don’t get any phone calls but we get our discharge, they have our discharge reports but no, we don’t speak directly to them. (Madison, provider)*

Increased communication following patient discharge was also recommended to improve transitions. Providers described the potential benefit of receiving ongoing feedback from primary care and community-based providers as patients transitioned out of hospital, particularly for those who received referrals (for services or facilities). This feedback would assist providers with knowing if the appropriate level of supports and services were provided, which would also benefit future patients who may experience similar challenges.

*So, if you don’t know where to send people or like you don’t know what they need to do to go to a certain place, and we also, we don’t really get any feedback on… if we’ve sent someone somewhere, unless they come back to the hospital and we can specifically ask, then we don’t know how it went or if that was appropriate or not… Well, [it would help] to know if it was an appropriate referral or not and why, would probably be a good start. I mean it’s hard to expect to know like how the person did, that would be nice to know, but you know. (Chloe, provider)*

## Discussion

Using qualitative methodology, we explored service recommendations made by patients, caregivers, healthcare providers, and decision-makers to improve experiences with transitions in care for older adults with hip fracture. To our knowledge, this is one of the few studies, that has explored in-depth patients’ and families’ hip fracture journeys, in addition to capturing perspectives from providers and decision-makers. Importantly, we identified opportunities for improvement across the continuum of care such as preventive, acute and rehabilitation care, and overall system navigation (particularly in the community) following the initial institutional care. Despite regional variation in hip fracture outcomes, the recommendations made by participants were similar. While we identified recommendations for different sectors (hospital, community) as well as transitions between them, the findings point to the need for targeted, integrated services with primary care at the centre. Due to the aging population worldwide, paired with the expected increase in the prevalence of hip fractures, we believe these recommendations would be relevant and applicable for further reflection to other health jurisdictions with different healthcare systems.

### Hospital-based recommendations

Hospital-based recommendations to improve experiences with transitions in care were centred around respect, communication, and staffing levels. Patients and caregivers described the importance of being treated as a unique person, rather than another patient with a hip fracture. Similarly, Baxter and colleagues identified ‘knowing the patient’ as a key recommendation in their qualitative research on safe transitions in care for older adults [30]. Specifically, participants in Baxter and colleagues’ study expressed the importance of getting to know patients beyond their medical condition to understand goals and expectations, living and psychosocial situations, as well as any potential fears about transitioning out of hospital [30]. In order to develop this understanding, it was important for staff to build trust and rapport with their patients. These collective findings are supported by a recent framework developed by Stolee and colleagues to support transitions in care for older adults with hip fracture, which highlighted the importance of patient involvement and choice, relationships between healthcare providers, patients, and families, documentation, and information sharing [31], all of which align with the fundamental principles of person-centred care [32].

Ongoing communication allows for the opportunity to develop trust between patients and healthcare providers [33] and it has been associated with better patient satisfaction [34–36]. Despite understanding the importance of communication and getting to know patients, acting on these recommendations and implementing them in practice often remains difficult. A key factor in supporting these recommendations is ensuring that hospitals are equipped with the appropriate resources, including adequate staffing (levels and roles). Previous research has identified associations between staffing levels (nurse to bed ratios) and both the quality and quantity of interactions between hospital staff and patients [37, 38]. Evidently, adequate staffing is the first step in helping to improve communication. However, it is important to better understand how staff time is spent and how care is provided. In addition to ensuring hospitals are equipped with appropriate resources, it is also important to consider the role patients and families play in improving communication. A systematic review examining communication skills training interventions identified that patients who receive training are more actively involved in their healthcare interactions [39]. With the increased focused on patient-centred care and shared decision making, the involvement of patients and families in improving the quality and quantity of interactions with healthcare providers is an important consideration. Ultimately, the goal is to identify how to facilitate authentic interactions, through the relational aspects of care, between staff and patients to enhance overall patient experiences in hospital and with care transitions.

### Community-based recommendations

A key finding from this study was the importance of community-based strategies through the identification of individuals who are at risk and proactive and preventative approaches. Previous research has shown almost half of patients with hip fracture visited the emergency department or were admitted to hospital in the year prior to their hip injury, with one quarter of these being fall-related [40]. In one particular study, of those who experienced a fall (*n* = 14), only five received information on falls prevention. Previous research has identified a history of falls as a risk factor for future falls [41, 42], further highlighting the importance of ongoing proactive, preventative, and outreach approaches in the community. Interventions addressing falls, including exercise and multifactorial programs (medication management, psychological interventions, environmental modification, physical intervention), have demonstrated a reduction in the risk of falls [43]. However, challenges remain for both healthcare providers and community-dwelling individuals. Community healthcare providers require tools that have been clinically validated to identify older adults at risk for falls [43] and community-dwelling adults need to be screened, referred to, and able to access these preventative programs. An opportunity is available to mitigate these challenges by embedding physical therapists as part of publicly funded, community primary health teams for assessment and treatment [44]. Physical therapists have the training and expertise to perform comprehensive physical and screening assessments, provide self-management support, and assist with health system navigation [45]; thus, highlighting the important role that could be played in injury prevention for older adults in the community.

Another opportunity to mitigate the challenges identified by participants in our study is to improve integrated primary and community-based care, which could be done through models such as the Guided Care Model [46]. The Guided Care Model is delivered by a registered nurse in a primary care office and combines principles from chronic care and self-management to improve access to care, self-care, and health outcomes. The role of the Guided Care Nurse is to complete comprehensive assessments, provide transitional care, develop action plans, monitor patient progress, provide individualized coaching, support and education, and assist with accessing community services [47]. This model has shown promising outcomes in improving quality of care and satisfaction with communication and reducing costs [47, 48].

### Cross-sectoral-based recommendations

Our findings also highlighted the importance of improved system navigation for patients and caregivers following hospitalization for a hip fracture. Patients and caregivers discussed the need for proactive navigation in terms of learning about available resources. Funk and colleagues proposed a multi-faceted, person-centred approach to improve system navigation issues consisting of: information dissemination and awareness of services, the creation of formal navigation support, and the integration of services [49]. Participants also recommended ensuring patients had consistent access to a care navigator to assist with information sharing and coordination of resources along the entire care journey. Care navigators provide individualized support to patients and are often responsible for advocating on behalf of the patient, coordinating care, supporting community engagement, completing needs assessments, providing education, emotional and psychosocial support, reducing barriers, and navigating services [50]. Care navigators are common practice with certain populations, such as individuals with cancer [51–54], persons with dementia [55, 56], and older adults [57, 58] and have demonstrated a number of positive outcomes, such as improved patient satisfaction [51, 53, 54], quality of life [52], and patient and family experiences [59]. Despite being common practice among some populations and the having the potential to improve outcomes, there are a number of factors that can support or impede the implementation of care navigators in practice [59]. Identifying an organizational need for care navigators, ensuring funding and resources are available, engaging a multidisciplinary team, outlining the care navigators’ roles, establishing workflow, providing training, supervision and mechanisms for communication, encouraging buy-in from stakeholders, and assigning appropriate caseloads were noted as factors supporting the implementation of care navigators [59]. In addition to these factors facilitating the integration of care navigators, it is important to consider the readiness to implement this role, including support from senior leadership, technological infrastructure to facilitate communication, partnerships across sectors, referral processes, and staff capacity [60]. With these considerations, it is also critical to ensure that individuals in this role would have time to spend with patients and families, instead of being consumed with burdensome administrative tasks [49]. Older adults with hip fracture often experience a number of transitions in care prior to reaching their final destination [61]. Additionally, due to the common intersection of hip fracture and cognitive impairment [62, 63], it is critical to further explore the potential role of care navigators for all persons who experience a hip fracture. Care navigators have been well integrated as part of the patient’s care team in other populations, so there is potential to use these as models for older adults with hip fracture.

Across all sectors and the transitions between them, patients and families should be proactively engaged as partners in their care. Providing the necessary supports and tools for patients to be empowered and engage in self-management activities could facilitate improved experiences with care transitions. Along the spectrum of healthcare, self-management activities can improve individuals’ health status by targeting problem solving, resource seeking, goal setting, and decision making skills [64]. For older adults with hip fracture, some of these self-management activities may include education around deconditioning and decline (physical and cognitive), shared decision making, and medication management in order to better prepare patients and families for life post-discharge.

#### Limitations

There are a few limitations of this research to be noted. First, our methods of data collection included both in-person interviews and telephone interviews, which had the potential to impact the data collected as non-verbal communication could not be observed for all participants. Despite this, telephone interviews have been identified as a method for collecting high quality responses [65]. Second, the majority of our patient and caregiver participants were White and English-speaking, it is possible that individuals from different ethnic or cultural backgrounds, or those who do not speak English as their first language, had differing or additional recommendations. This is an area that warrants additional research. Third, we required consent to contact patients and caregivers, which was obtained from healthcare providers. Some patients and caregivers declined contact with the researchers, and it is possible that they had differing experiences, perspectives, and recommendations that were not captured. Fourth, the interview guides were not co-developed with patients and caregivers, so it is possible that we did not fully capture what mattered to patients and caregivers about their care transitions.

## Conclusions

Transitions in care are a complex and vulnerable time for patients and caregivers. We identified a number of actionable recommendations based on interviews with patients, caregivers, healthcare providers, and decision-makers. Hospital-based recommendations focused on improving the consistency, frequency, and comprehensiveness of communication between hospital providers and between providers and families, increasing staffing levels, and treating patients and families with respect. Community-based recommendations included identifying at-risk individuals early and providing preventative and educational programs. Cross-sectoral based recommendations were grounded in enhanced system navigation. Overall, our findings highlighted a clear need for community-based integrated care, specific to individuals with hip fracture, with primary care playing a central role. Addressing and acting on these recommendations should be a priority, as they have the potential to improve experiences, and possibly health outcomes for older adults with hip fracture.

## Data Availability

The datasets generated and/or analyzed during the current study are not publicly available in order to protect participant identity, but are available from the corresponding author upon reasonable request.

## Abbreviations

IRU: Intensive Rehabilitation Unit
PT: Physical Therapist
OT: Occupational Therapist
PSW: Personal Support Worker
